# Using Statistical Modeling for Enhanced and Flexible Pharmacovigilance Audit Risk Assessment and Planning

**DOI:** 10.1007/s43441-020-00205-4

**Published:** 2020-08-17

**Authors:** Min Zou, Yves Barmaz, Melissa Preovolos, Leszek Popko, Timothé Ménard

**Affiliations:** grid.417570.0 0000 0004 0374 1269 F. Hoffmann-La Roche AG, 4070 Basel, Switzerland

**Keywords:** Quality assurance, Pharmacovigilance, Drug safety, Statistical modeling, Good pharmacovigilance practice (GVP), Audit

## Abstract

**Background:**

The European Medicines Agency Good Pharmacovigilance Practices (GVP) guidelines provide a framework for pharmacovigilance (PV) audits, including limited guidance on risk assessment methods. Quality assurance (QA) teams of large and medium sized pharmaceutical companies generally conduct annual risk assessments of the PV system, based on retrospective review of data and pre-defined impact factors to plan for PV audits which require a high volume of manual work and resources. In addition, for companies of this size, auditing the entire “universe” of individual entities on an annual basis is generally prohibitive due to sheer volume. A risk assessment approach that enables efficient, temporal, and targeted PV audits is not currently available.

**Methods:**

In this project, we developed a statistical model to enable holistic and efficient risk assessment of certain aspects of the PV system. We used findings from a curated data set from Roche operational and quality assurance PV data, covering a span of over 8 years (2011–2019) and we modeled the risk with a logistic regression on quality PV risk indicators defined as data stream statistics over sliding windows.

**Results:**

We produced a model for each PV impact factor (e.g. 'Compliance to Individual Case Safety Report') for which we had enough features. For PV impact factors where modeling was not feasible, we used descriptive statistics. All the outputs were consolidated and displayed in a QA dashboard built on Spotfire^®^.

**Conclusion:**

The model has been deployed as a quality decisioning tool available to Roche Quality professionals. It is used, for example, to inform the decision on which affiliates (i.e. pharmaceutical company commercial entities) undergo audit for PV activities. The model will be continuously monitored and fine-tuned to ensure its reliability.

## Background

Preventing harm from adverse reactions in humans arising from the use of authorized medicinal products and promoting the safe and effective use of medicinal products are the fundamental objectives of pharmacovigilance (PV). Marketing Authorization Holders (MAH) are required to implement and maintain a quality system to fulfil their PV activities [1]. Activities required to monitor the performance and effectiveness of the pharmacovigilance system and its quality system include PV audits. The European Medicines Agency Good Pharmacovigilance Practices (GVP) guidelines provide a framework for PV audits and emphasize that a risk-based approach to PV audits should be applied. Beside the requirement to have strategic and tactical PV audit planning, GVP Module IV provides very limited guidance on the methods to be used for risk assessment [2].

PV audits can be performed for different entities i.e. vendors, central process, business partners and affiliates (i.e. pharmaceutical company commercial entity) [2]. PV affiliate audits usually represent the majority of audits performed by large and medium size pharmaceutical companies, have a broad scope, are long (the industry benchmark is around 3 to 5 days on site) and require significant resources (2–6 auditors). Hence in this project the focus has been PV in the context of an affiliate audit.

Quality Assurance (QA) teams of large and medium sized pharmaceutical companies generally conduct annual risk assessments for PV, based on retrospective review of data and pre-defined impact factors to plan for PV audits [3–6]. Conducting an annual risk assessment requires a high volume of manual work and mobilizes QA resources. This is a reactive process in that audits are executed based on risk assessed from past data (from several months up to a year). For large and medium sized companies, auditing the entire “universe” of individual entities on an annual basis is generally prohibitive due to sheer volume, placing an even greater emphasis on a sound and timely risk assessment strategy to ensure QA activities are prioritized to assess the identified risks contemporaneously. Further complicating the picture, there is not an industry-standard set of parameters for conducting a QA risk assessment of PV entities. As such, the impact factors are often defined arbitrarily and should be considered as subjective [7].

A risk assessment approach that enables efficient, flexible, focused and targeted PV audits is not currently available. However, the industry has recently been trying to leverage modern developments in data management and the IT systems that facilitate the cross-analysis of PV and clinical trial data. Clinical statistical analysis can be performed on this data to help identify specific areas of risk that can be further assessed via a QA activity (e.g. audit) or in some instances, highlight areas that would benefit from immediate mitigating interventions, with the aim of preventing occurrence and/or recurrence of audit findings.

We propose a model that will provide insight to QA professionals to assess PV quality risks more holistically, timely and efficiently, as well as enable more risk-targeted audit planning and preparation, with the aim to directly contribute to the assurance of patient safety in the post-authorization setting. The model is currently intended to supplement the previously existing risk assessment approach, which includes a review of discrete factors such as time since previous audit, outcome of previous audit, organizational structure, self-reported deviations from procedural documents and a review of compliance against key PV processes (“key PV processes” are identified and defined during strategic and tactical planning phases as those processes/process outputs that have significant or direct impact to patient safety and the ability to maintain a complete and up-to-date benefit-risk profile for the products). The outputs of the model and the review of descriptive data as described above are then assessed together to determine the audit program at an individual audit level.

The development of a statistical model that can help assess PV quality risks requires a deep understanding of data science, pharmacovigilance and QA. The project has been conducted by the Roche quality analytics and insights team, a team of data scientists and business analysts, in collaboration with Roche PV and QA subject matter experts (SMEs). The mission of the Roche quality analytics and insights team is to build data-driven solutions for QA at Roche to complement and augment traditional QA approaches to improve the quality and oversight of GVP- and Good Clinical Practices (GCP)-regulated activities.

## Methods

### Prerequisites

To estimate PV quality risk for affiliates, we relied on the outcomes of previously conducted audits. There was a potential bias in this approach as affiliate audits were performed for each entity at irregular intervals following decisions to audit based on varying criteria. However, we had a data set covering a span of 8 years where for the years 2013–2014 each individual affiliate had been audited at least once.

The quality assurance data (i.e. individual quality issues) reported through an audit were labeled with categories, sub-categories and finding statements. The source database was the Roche audit finding management tool. To translate the quality issues into areas that could be interpreted across sponsors (while directly linking to key GVP requirements), we mapped all the individual findings statements to defined PV impact factors (PV IF). The consolidated list of PV IF considered in our analysis is described in Table 1. See Table 2 for examples of mapping of finding statements to PV IF.

**Table 1:** PV impact factors

Area	PV impact factor
Safety data acquisition, management and submission	Compliance to Individual Case Safety Report (ICSR) process
Communication, Implementation and Quality Management of Risk Minimization activities	Compliance to risk minimization activities
Communication, distribution and quality management of Direct Healthcare Professional Communications (DHPC) activities	Compliance to DHPC activities
Communication, implementation, distribution and quality management of reference safety information	Compliance to safety updates to local labels

**Table 2:** Individual finding statements (already utilized in the company’s audit and finding management system) mapped to the PV IF

PV impact factor	Example of individual finding statements
Compliance to ICSR process	ICSR receive dates were inaccurately determined and/or recorded
	Follow-up on ICSRs was not performed, untimely and/or not documented
	Processes and/or procedures for case identification and collection of potential AEs were not defined, inadequate and/or not followed
	Reconciliation/case transmission verification with all relevant internal functions was not performed, untimely, inadequate and/or not documented
Compliance to risk minimization activities (RminAs)	Communication and/or submission of Risk Management Plans (RMPs) or additional RMinAs to the regulatory authority was not completed, untimely and/or not documented
	There was a lack of oversight for local implementation of PV activities or RMinAs
Compliance to safety updates to local labels	Procedures and/or processes for updating reference safety information were not defined, inadequate and/or not followed
	Promotional material was not updated with new safety information or not updated in a timely manner

Quality risk modeling for PV audits has a solid business use case, hence it was essential that the resulting model was interpretable and that the identified impact factors were usable for our stakeholders, i.e. Roche Quality PV Strategy. However, considering the availability and quality of the data, and the involvement of certain subjective variables such as Health Authority (HA) engagement, the team decided to exclude the following PV IFs from the modelling exercise and utilized the descriptive analytics to support risk assessment decisions:

- Compliance to Post Authorization Safety Study (Interventional and Non-interventional) requirements for locally managed studies
- Compliance to Local HA Commitments
- Adequate implementation of the Corrective Action Preventive Action (CAPA) process
- Adequate oversight of local business partners to ensure compliance with PV requirements

For each PV IF, we conducted in-depth business process analysis and specified sub-impact factors and the related data topics for further identification of the data sources.

### Data

We collaborated with global process owners and data SMEs from the relevant Roche safety, regulatory and commercial functions to identify data attributes which are relevant for addressing the identified data topics. This led us to gather relevant raw data from Roche safety (Adverse Event Management, Pharmacovigilance Agreement Management, Pharmacovigilance Master File, Risk Management System), Clinical Study Management, Commercial, Marketing Research and Patient Support Programs, Quality Management and Regulatory Labelling Management databases. Our quality data set consisted of 1171 individual findings collected over 8 years, which had been reported from affiliates audits between the years 2011–2019. On average, 24 audits and 2 inspections were conducted each year, with each individual affiliate being audited 2 times over the defined time period. A typical audit or inspection generated around 6 findings on average.

### Features

We translated the business data topics to quantitative features for model fitting. For example, to assess the risk associated with the quality of data entered in the Safety Receipt System sub-impact factor, we identified how much error was captured during Individual Case Safety Report (ICSR) safety processing database quality review process which was not captured during the earlier review in the receipt system as one of the business data topics. This data topic led us to further explore the number of cases cycling in the ICSR safety case processing database workflow, the number of cases updated in the safety case quality review and ratio of key data attributes being corrected in the safety processing system (for example: seriousness, pregnancy assessment and primary source) as features. Following such an approach, we generated around 50 data topics and 85 quantitative features. See Table 3 for an example on how the PV IF ‘Compliance to ICSR process’ has been translated into sub-impact factors, data topics and features for the model.

**Table 3:** Data topics translated to model features, e.g. of compliance to ICSR process

PV sub-impact factor	Data topics	Translated features (calculated per affiliate per time window)
Have we identified AEs which should have been reported to Roche from all sources?	Compare the volume of units sold and number of AEs reported per Affiliate per time period. Any obvious changes over time	Cross time window ratio of total number of AEs reported to sum of units sold
	If we have active local studies which have been created for some time but no reported AE, assess the risk of AE under-reporting	Ratio of average number of active studies without AEs reported to total number of active studies
	Are there obvious changes over time in the AE reporting rate per primary source?	Ratio of total number of AEs from a given primary source to total number of AEs from all primary sources
ICSR receipt and processing timeliness	Timeliness of case transfer to the local processing center	Ratio of number of cases transferred late to total number of cases transferred to the local processing center
	Timeliness of case processing in the safety receipt system	Ratio of number of cases processed late to total number of cases processed in the safety receipt system
	Timeliness of case disposal from safety receipt system to processing system	Ratio of number of cases disposed late to total number of cases disposed to the processing system
Quality and timeliness of submissions	If there is high proportion of unjustified nonsubmission cases, assess the risk of identifying findings in ICSR submission	Ratio of number of nonsubmission cases with unjustified reason to total number of nonsubmission cases, calculated by health authorities and license partners cases separately
	How have we compliant with the local submission timelines?	Number of days late according to the local submission timeline by quantiles
	Timeliness of case submission to health authorities or license partners	Ratio of number of cases submitted late to total number of cases submitted to health authorities or license partners

### Modeling Approach

To model risk, we followed the GVP guidelines (Section IV.B.2), “*where risk is defined as the probability of an event occurring that will have an impact on the achievement of objectives, taking account of the severity of its outcome and/or likelihood of non-detection by other methods*” [2]. These events are typically recorded as audit and inspection findings or self-reported deviations. The main challenge is the subjectivity in these procedures, for instance two auditors might have different assessments of a finding being minor or major, and it is hard to decide if an audit that results in five or six minor findings is worse than one that results in a single major finding. For these reasons, we decided to focus the modeling on the binary outcome of an audit, finding vs. no finding, and leave it to the PV Quality professionals to further assess the situation among the affiliates flagged as potentially at risk.

In the binary outcome modeling approach, the risk is defined as the probability *p* of getting a finding, should one conduct an audit, and past audits provide data on the realizations of these binary random variables (technically a Bernoulli(*p*) random variable). The risk is never observed, though, unlike the actual outcome of an audit, so we needed a model to estimate it. One way to do so is to specify risk covariates based on the input of quality professionals, as described in ‘Prerequisites’, and fit a logistic regression on these risk covariates to the audit outcomes. The output of the regression then provides an estimate of the risk.

Quality strategists traditionally base their assessment of PV processes on PV data collected in the past 12 to 18 months. This data usually takes the form of streams of individual events such as the reception of an adverse event report, from which strategists extract risk metrics in the form of summary statistics. We automated this process on historical data by computing these statistics on sliding windows, much like when computing a moving average, and we obtained a multivariate time series of risk covariates for every affiliate. We still had to apply a logistic model on these time series to get time-varying risk levels. To determine the parameters of such a model, we used the fact that past audits correspond to single points in these time series, so we fitted a logistic regression on the values of the risk levels at these time points.

Finally, we had to choose the resolution of the model. At the two ends of the spectrum, we could have picked a single model to predict the occurence of any finding, or we could have built one model per standard finding statement. The former would not have been very informative, by mixing information from unrelated categories, and the latter would have lacked statistical robustness due to the rarity of certain findings. We thus decided to build one model per impact factor (or sub-impact factors in the case of impact-factors involving larger numbers of findings) introduced in ‘Prerequisites’, and include in the model of a given impact factor only the features related to it.

### Example

We illustrate this methodology with the PV IF “Have we identified AEs which should have been reported to Roche from all sources?”. For every affiliate, we constructed the time series of related risk covariates listed in Table 3. To find the values of these time series at a given timestamp, we collected relevant data points in a 24 months window prior to it and computed various summary statistics. The ratios of certain categories of reported AEs to all reported AEs in that window can indicate different patterns of safety reporting among affiliates and across time. In these special categories, we considered spontaneous AEs, AEs from literature, AEs from clinical studies and AEs from non-interventional studies. The ratios of reported AEs to sales volumes can indicate if safety reporting volume follows drug usage, and comparing these statistics to the same ones collected in a 6 months window can indicate a change of reporting patterns. Finally, the ratio of invalidated AEs to all reported AEs can be indicative of how stringent an affiliate is at reporting suspicious cases. The use of ratios in these statistics rather than extensive values allowed to compare affiliates of different sizes to each other.

## Results

**Fig. 1:**
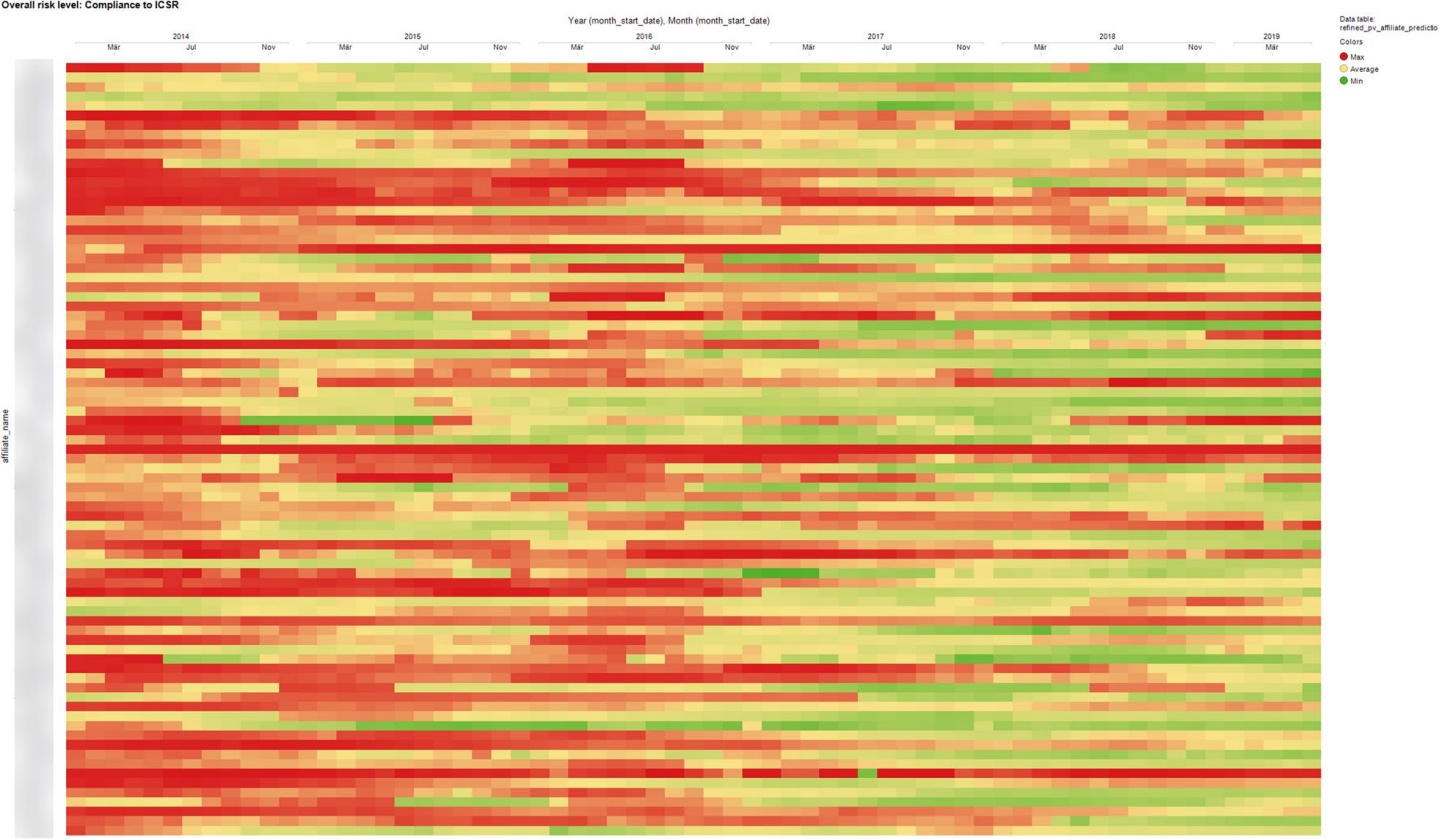
Model output for a given IF visualized with Spotfire^®^

We built a visual and interactive dashboard using Spotfire^®^. Figure 1 displays the estimated risk for a given PV IF across Roche affiliates from January 2014 to May 2019. Each unit on the y-axis represents a Roche affiliate and each unit on the *x*-axis represents a month. A cell on the heatmap represents the estimated risk for a given PV IF for the selected Roche affiliate and month. The gradient color is automatically displayed based on the cell value and color scheme which has three anchor points: Min, Average, and Max automatically calculated for each PV IF. The colors at these points are set to green, yellow, and red respectively, which means that the color gradient shifts from green to yellow to red, dynamically based on the cell value. This visualization enables Roche Quality professionals to quickly assess the overall risk for a given PV IF across affiliates and to observe any obvious change overtime.

Due to data availability and quality limitations in some continuously developing process areas, we were not able to generate all desired features for the identified data topics. For example, we identified a number of data topics related to Compliance to Direct Healthcare Professionals Communications (DHPC) activities Impact Factor. After further analysis, we were only able to produce one feature to assess the timeliness of DHPC distribution to healthcare professionals. To address challenges like this when we could not generate enough indicative features for modelling, we visualized the available features across affiliates and time windows to assist Roche Quality professionals to evaluate the risk for the related PV Impact Factors together with other sources of intelligence.

## Discussion

The output and performance of the model were satisfactory for our use case. The model outputs were used as part of a comprehensive risk assessment strategy to determine the Affiliate audit sample. Due to the replacement of part of the manual risk assessment to the model-based approach, a primary benefit has been enabling Roche Product Development Quality to run the risk assessment on a quarterly basis (vs. annual basis), with an added benefit of reallocating FTEs to other strategic priorities and greater sustainability from a resourcing perspective. As noted above, the model output is not the sole driver for audit selection, but rather contributes to a wider set of data points that drive the sample selection. As such, the model is best classified as Quality Decision Support Tool—part of a broader effort to use data and statistics to enhance quality assurance activities [8, 9]. In addition, the temporal nature of the model outputs enables a quality assurance program that is more targeted toward the current/future potential compliance issues rather than a retrospective approach.

### Limitations

As data came from various systems and had different standards (see also ‘Data’), the process for collection, cleansing and wrangling was time consuming but in line with typical data science projects [10]. We could not produce a model for certain PV IF (see ‘Prerequisites’) due to incomplete data (e.g. no data to reflect qualitative information such as AE reporting "culture") or because the signal to noise ratio was low. In such circumstances, using descriptive statistics was more informative, hence we made tradeoffs (with the guidance of our PV SME) between the value of developing a model versus deriving insights from visualizing the data.

As mentioned in ‘Prerequisites’, there was a potential bias in our approach as not every affiliate had been audited at the same frequency, with the exact same scope and the same number of times. Thus, any risk that we could estimate excludes risks that had not been regularly detected in the past.

Our model was developed using data that were generated according to the Roche processes and standards. The data (e.g. volume of ICSRs) were also reflective of the Roche product portfolio. Further validation (using high level principles of our model) on other pharmaceutical companies data could be feasible but would require cross-company collaborations and data sharing like in the GCP arena [11].

We had planned for a periodic review of audit outcomes versus model predictions in order to fine tune the model. However, due to COVID-related restrictions impacting the ability to conduct audits in the early-mid part of 2020, we have not yet had the opportunity to initiate this analysis.

Last but not least, we would like to caution that it is a tool to support decision making hence not replacing human judgment and Roche QA colleagues remain fully accountable for selecting a particular entity to audit.

## Conclusion

In this paper, we proposed a statistical model to enhance the risk-based approach for PV affiliate audits. Using a straight forward modeling approach (i.e. logistic regression), descriptive statistics and user-friendly visualization, we developed a holistic, efficient and objective risk assessment tool. It has been implemented and is used by PV Strategy within the Roche Product Quality organization for quality decisioning in the affiliate audit PV space. Of note, the model will be continuously monitored and fine-tuned to ensure its reliability. This project is part of a broader effort at Roche Product Quality to leverage advanced analytics to augment and complement traditional QA approaches.
